# Single cut distal femoral osteotomy for correction of femoral torsion and valgus malformity in patellofemoral malalignment - proof of application of new trigonometrical calculations and 3D-printed cutting guides

**DOI:** 10.1186/s12891-018-2140-5

**Published:** 2018-07-11

**Authors:** Florian B. Imhoff, Joscha Schnell, Alejandro Magaña, Theresa Diermeier, Bastian Scheiderer, Sepp Braun, Andreas B. Imhoff, Robert A. Arciero, Knut Beitzel

**Affiliations:** 1 Department of Orthopaedic Sports Medicine, Technical University Munich, Ismaninger Str. 22, 81675 Munich, Germany; 2 Department of Orthopaedic Surgery, University of Connecticut, 263 Farmington Ave, Farmington, CT 06030 USA; 3 Department of Mechanical Engineering, Institute for Machine Tools and Industrial Management, Technical University Munich, Boltzmannstr. 15, 85748 Garching, Munich Germany

## Abstract

**Background:**

The purpose of this study was to perform a derotational osteotomy at the distal femur, as is done in cases of patellofemoral instability, and demonstrate the predictability of three-dimensional (3D) changes on axes in a cadaveric model by the use of a new mathematical approach.

**Methods:**

Ten human cadaveric femurs, with increased antetorsion, underwent a visually observed derotational osteotomy at the distal femur by 20°, as is commonly done in clinics. For surgery, a single cut osteotomy with a defined cutting angle was calculated and given using a simple 3D-printed cutting guide per specimen, based on a newly-created trigonometrical model. To simulate post-operative straight frontal alignment in a normal range, a goal for the mechanical lateral distal femur angle (mLDFA) was set to 87.0° for five specimens (87-goal group) and 90.0° for five specimens (90-goal group). Specimens underwent pre- and post-operative radiographic analysis with CT scan for torsion and frontal plane x-ray for alignment measurements of mLDFA and anatomical mechanical angle (AMA).

**Results:**

Performed derotation showed a mean of 19.69° ±1.08°SD (95% CI: 18.91° to 20.47°). Regarding frontal alignment, a mean mLDFA of 86.9° ±0.66°SD (87-goal-group) and 90.42° ±0.25° SD (90-goal group), was observed (*p* = 0.008). Overall, the mean difference between intended mLDFA-goal and post-operatively achieved mLDFA was 0.14° ±0.56° SD (95% CI: -0.26° to 0.54°).

**Conclusion:**

A preoperative calculated angle for single cut derotational osteotomy at the distal femur leads to a clinically precise post-operative result on torsion and frontal alignment when using this approach.

## Background

Increased femoral antetorsion is one important risk factor for patellofemoral instability and anterior knee pain syndrome in teenagers and young adults [1–4]. Correction osteotomy at the distal femur has been shown to be a reliable option for correction of torsional pathologies [4–8]. Increased lateral facet pressure and increased medial retinaculum strain was shown to be correlated with increased femoral antetorsion [9–11]. In the author’s clinical observation, valgus deformities often appear in conjunction with torsional deformities in cases of patellofemoral instability. Valgus malalignment is defined as a lateral deviation of the mechanical axis (line from the femoral head to the upper ankle joint), which is referred to the center of the knee joint on a frontal plane radiograph [12]. For more accuracy and confirmability, the amount of valgus deviation is usually measured as the angle between the mechanical axis of the tibia versus the mechanical axis of the femur [13, 14]. Regarding the femur, mechanical and anatomical axes differ on a frontal plane radiograph, whereas the mechanical anatomical angle (AMA) depends on the femoral torsion [15]. While changes of the mechanical and anatomical axes are likely to occur in derotational osteotomy, unintended changes on axes should be minimized. According to previous publications, rotational osteotomies may result in unplanned three-dimensional (3D) effects, such as aggravation of valgus malalignment [1, 16].

Thus, a reliable anatomical reference of the cutting plane is the most important surgical step to prevent these complications. Mathematical models, commonly used in robotics for calculating transformations for serial kinematics, allow for a prediction of the resulting 3D changes when rotation of a limb is performed with a defined angle of the rotation-joint [17]. When these models are transferred to derotational osteotomies, prediction of the exact changes of axes can be made when there is a known angle of the cutting plane and the tubular bone is rotated on its axis. However, these calculations are not easily applicable in the OR. Therefore, easy to handle tools, such as pre-calculated tables and individual cutting guides, could help surgeons increase the precision of their surgical procedure.

The purpose of this study was to perform a derotational osteotomy at the distal femur, as it is done in cases of patellofemoral instability, and demonstrate the predictability of three-dimensional (3D) changes on axes in a cadaveric model. We hypothesized that a distal femoral derotational osteotomy with a defined inclination of the cutting plane can correct the frontal alignment due to correction of torsion according to the preoperative calculated values.

## Methods

### Experimental design

For proof of concept, a standardized protocol was developed to investigate mathematical predictions in a clinical cadaver model. Human cadaveric femurs with individual increased antetorsion and valgus imitating mechanical axes, were radiographically analyzed for exact angle calculations. A derotation of 20° was defined across all specimens, which in terms of consistency is clinically applicable [2, 4]. In this femur cadaver model, it was not possible to obtain the corrective goal on a total leg axis to achieve a straight frontal alignment, as it would be done in clinical practice. Therefore, the goal for frontal alignment correction was based on the femoral mechanical axis, and the mechanical lateral distal femur angle (mLDFA) was set to mLDFA = 87.0° for five specimens (87-goal group), and mLDFA = 90.0° for five specimens (90-goal group). These angles were chosen as they represent normal range angles and allow for statistical comparison of two groups within a clinical significant 3° difference [15]. Femurs were assigned to a group, based on their preoperative mLDFA, to achieve homogenous corrective angles for both groups. Based on a new trigonometrical model for single cut rotational osteotomy, tables with practical values were created for future implementation into clinical use. Then, an individual defined cutting angle from a lateral approach was given for each specimen to correct rotational and frontal axis. This unique inclined single-cut was provided by a simple 3D-printed cutting guide. Postoperatively, achieved angles were measured radiographically and compared to their intended goal.

### Specimens

Ten cadaveric femurs, five left and five right, were used in this study, which had been obtained from MedCure (MedCure, Inc., Cumberland, RI, USA). Specimens had served for an Orthopaedic Residency Training Program for total hip (THA). Each femur was dissected free from all its soft tissue and muscle, and all implants were taken out. Femoral heads, which were previously removed for THA, were then fixed with k-wires on the corresponding femoral neck with an individual increased antetorsion (25° - 45°) with regards to clinical observed values [2–4]. At the distal femur, joint lines were artificially cut oblique from lateral to medial to create a slight valgus malalignment, which was measured as a decreased mLDFA. Next, these prepared specimens underwent radiographic analysis. This study was reported to the institutional review board (IRB). It was documented that de-identified specimens do not constitute human subjects research, and no IRB approval was required.

### Radiographic imaging

If increased maltorsion is suspected in clinics, magnetic resonance imaging is usually performed to avoid additional radiation. However, attention must be given to different techniques and thresholds [18]. Due to the biomechanical nature of this proof of concept study, computer tomographic (CT) scans were performed using a standardized bone protocol preserving 3 mm axial slices (Osteo window b50s kernel algorithm, Siemens Somatom Definition Dual Energy 64 slice). For x-ray imaging, specimens were clamped on a x-ray-grit in a supine position, making sure that the distal femoral condyles were levelled perpendicular to the path of rays. Antero-posterior radiographs were taken with a C-arm (GE Medical Systems Inc.) and, according to the x-ray-grit, precisely combined to generate a panoramic view of the entire femur. CT scans, x-ray and measurements were repeated postoperatively. Measurement of the angles were repeated three times by one observer and the average was taken.

### Imaging analysis

Torsion measurement of the femur was done as described by Waidelich et al. [19], a CT-based method, which is used according to several clinical publications [4, 20, 21]. The center of the femoral head was determined on one image and the center of the greater trochanter was marked with an ellipse on a second image. The third image showed a tangential line at the posterior condyles. When all three images were merged, the positive angle between a line from the femoral head to the trochanter versus the posterior condyles equaled femoral antetorsion.

**Fig. 1:**
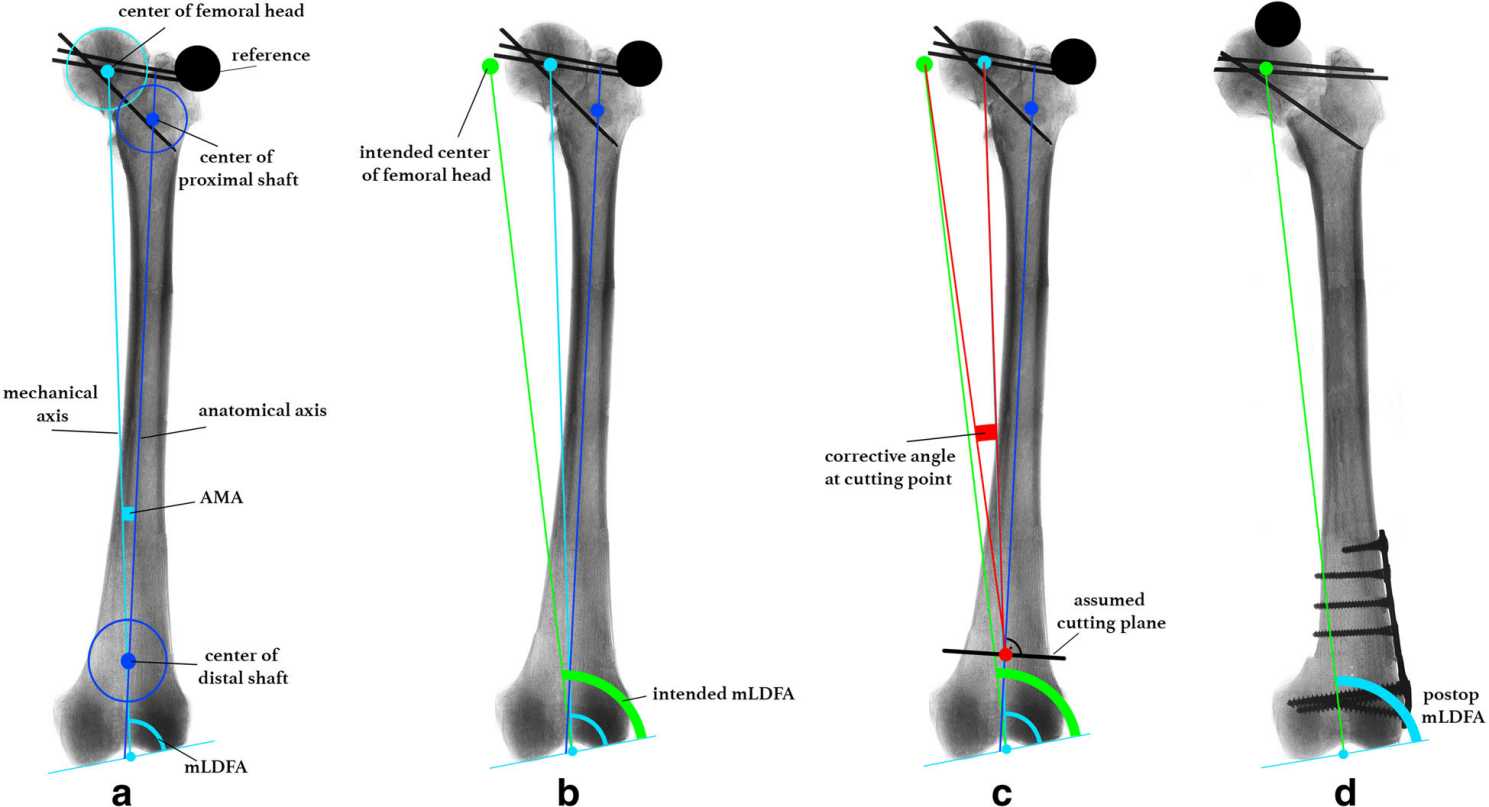
Planning of correction of frontal alignment (x-ray-grit is vanished for better visualization): **a** preoperative measurements and reference; **b** drawing of intended mechanical axis/center of femoral head; **c** measuring corrective angle at assumed cutting plane; **d** postoperative result

The frontal plane x-ray provided measurement of mLDFA and AMA according to Strecker [13]. The mechanical femoral axis is a line from the center of the femoral head to the center of the distal femoral joint line (Fig. 1a). The anatomical axis was drawn as a line from the middle of the proximal femur shaft to the middle of the distal shaft. This point was defined 7 cm from the distal joint line, where the cutting plane of the distal femoral osteotomy was assumed. Usually, anatomical and mechanical axes cross at the middle of the condyles and not at the joint line [12]. The angle between the anatomical and mechanical axis (AMA) was measured. The mechanical lateral distal femur angle is the angle between the mechanical axis and the distal femoral joint line.

In order to decrease valgus alignment, the femoral head was virtually planned to be medialized (Fig. 1b). As a result, mLDFA increased as well. The intended postoperative position of the femoral head was set in a manner that resulted in a mLDFA of 87.0°, and 90.0°, respectively. Then, the angle between the center of the femoral head, middle of the assumed cutting plane, and intended center of the femoral head was measured. This angle is also known as the corrective angle at the cutting point (Fig. 1c) [13]. A DICOM Imaging software (OsiriX Lite, PIXMEO SARL, Switzerland) was used for all measurements. Measurements and planning was done by the first author. Postoperative measurements were done at two different time periods with 6 weeks in between and average values were taken. All measurements were made to the tenth of a degree.

**Fig. 2:**
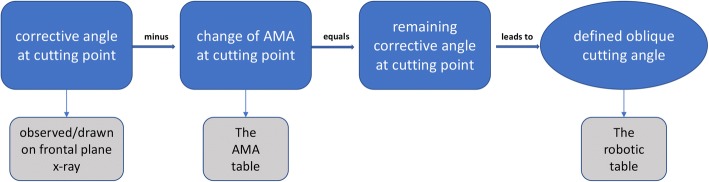
Calculation for clinical practice: Processing radiographically observed corrective angle and change of AMA to the remaining corrective angle, which leads to a defined oblique cutting angle

The following calculation had to be done to receive the remaining corrective angle (Fig. 2): Torsion angle and intended derotation angle changed the AMA, which was observed on the frontal plane x-ray, by a certain amount. This change of the AMA was subtracted from the corrective angle at cutting point, which resulted in the remaining corrective angle at cutting point. In order to achieve the amount of the remaining corrective angle on the frontal plane by means of a derotation, the cutting plane had to be inclined from a sagittal view. The precise value was determined using a trigonometrical formula, obtained from the robotics.

### Mathematical model

**Fig. 3:**
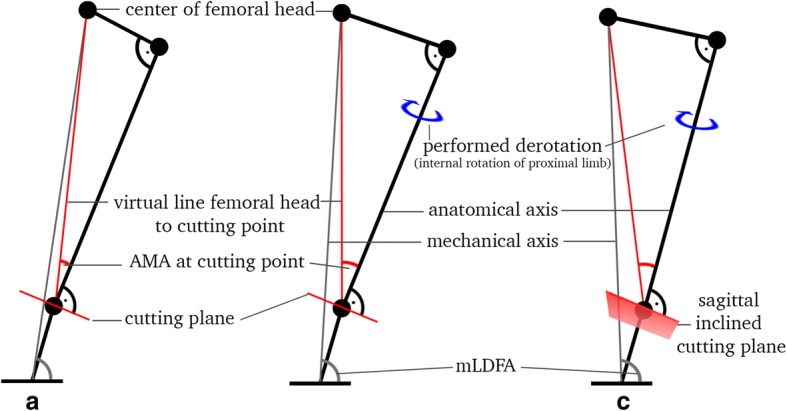
Elementary mathematical approach: **a** Increased antetorsion, decreased mLDFA; **b** If cutting plane is perpendicular, derotation leads to normal antetorsion and slight increased mLDFA; **c** If the cutting plane is oblique from a sagittal view, derotation leads to normal antetorsion and significant increased mLDFA

Derotation is defined as an external rotation of the distal limb, which equals an internal rotation of the proximal limb. The Pythagorean Theorem was used to calculate angular changes between the anatomical and mechanical axes of the femur. Measurement of the AMA on a coronal view at different torsion angles will result in a change of AMA when a derotation is performed (Fig. 3a, b). Then, an approach commonly known from robotics was applied [17]. The Denavit-Hartenberg transformation matrix calculates in a cartesian coordinate system (XYZ) the sagittal and coronal changes of axis when defining an oblique cutting angle on one plane and a rotation through its central axis (Fig. 3c). For transition into clinical practice, an inverse equation was derived: Intended change of axis on the coronal view and planned rotation equals an individual inclined cutting plane from the sagittal view. The mathematical model calculated change on frontal, sagittal, and axial axis. Trigonometrical formulas were processed with Matlab (MathWorks, Version R2017a) and Mathematica (Wolfram, Version 11.1).

### 3D printed cutting guide

In order to provide a precise cutting angle in a clinical setup, a new simple cutting guide was self-designed using open-source parametric 3D modeling software (FreeCAD, Version 0.16). The basic concept of the guide was to provide the exact cutting angle from a lateral approach and did not have to be adapted to the individual bone when overall orientation was maintained. This cutting guide provides two fixation holes for 2.0 mm k-wires and a proximal spacer to adjust the guide to the shaft in line with the anatomic axis on the coronal plane. A guiding slot on the lateral side was virtually mounted on top, allowing for the use of an oscillating saw. For each specimen, an individual cutting angle needed to achieve the proposed axis effect was imported to the designed guide. Printing of the common standard tessellation language files (*.stl) for each individualized guide was performed on a 3D printer (Ultimaker2+ extended, Ultimaker B.V., Netherlands) using simple PLA (polyactic acid) material.

### Surgical technique

**Fig. 4:**
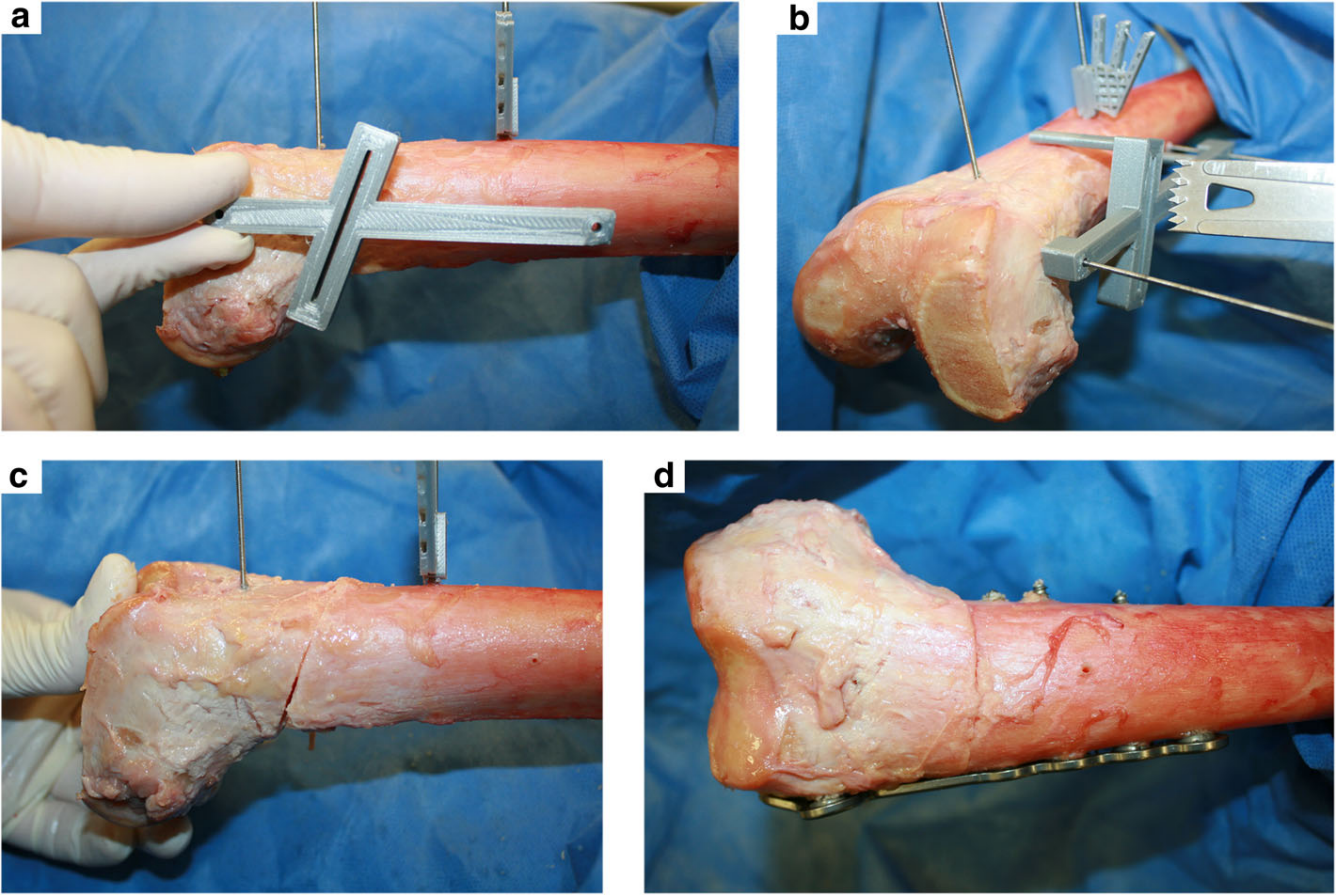
Surgery of specimens: **a** Cutting guide aligned parallel to the virtual shaft axis (distal: middle of the shaft; proximal: middle of the shaft at height of the greater trochanter), **b** Single cut osteotomy through the cutting guide, **c** Derotation by 20°, **d** plate fixation, resulting in slight varus change on the coronal axis

Surgical procedure specimens were clamped in a supine position on a table. For rotational osteotomy from the lateral side, the entry point of the single cut was set at 70 mm proximal to the knee joint line with regards to the plate design. A 3D-printed cutting guide was then fixed with two 2.0 mm k-wires on the lateral side of the femur (Fig. 4a). The guide was aligned parallel to the virtual anatomic axis from a coronal view and parallel to the virtual anatomical axis from a sagittal view. The virtual anatomical axis is a line from the middle of the proximal shaft at the greater trochanter to the middle of the distal shaft at the assumed height of the cutting plane, on a frontal and a sagittal view [22]. For rotational control, two 2.5 mm k-wires were inserted from anterior to posterior, one proximal to the assumed osteotomy and one on the distal side. An oscillating saw provided the perfect single cut with an individualized oblique angle (Fig. 4b, c). The cutting guide was removed, followed by derotation of 20° (external rotation of the distal limb). This was observed with a goniometer from an axial perspective regarding two k-wires, as is routinely done in clinical practice [23]. Osteotomies were fixed with a lateral distal femur plate (Fa. Arthrex, Naples, USA) (Fig. 4d).

### Statistical analysis

A 95% confidence interval with limits of ±1° was deemed to be clinically appropriate. A standard deviation of 1° in alignment correction, as observed with goniometric measurement, was assumed across specimens. Based on this desired level of precision using these estimates, a sample size calculation was performed which determined that at least eight specimens were required. Descriptive statistics including mean, range, and standard deviation (SD) were calculated to characterize the specimens. The results of the analysis are presented as mean values with corresponding 95% confidence intervals. Differences between pre- and post-operative measurements were analyzed with the Wilcoxon sign rank test, the non-parametric equivalent of the paired *t* test. The alpha level was 0.05 for all statistical tests. The analysis was conducted with STATA (Stata/IC 14.2, StataCorp LP).

## Results

### Radiographic outcome and statistical analysis

Pre-operatively, specimens showed mean antetorsion of 37.1° (range 27.6° - 48.7°) and a mean mLDFA of 84.5° (range 80.4° - 87.5°). Post-operatively, mean performed derotation was 19.69° ± 1.08°SD (95% CI: 18.91° to 20.47°). Post-operative frontal alignment in the 87-goal group was observed as a mean mLDFA of 86.9° ± 0.66°SD, and a mean mLDFA of 90.42° ± 0.25° SD in the 90-goal group. Overall, difference between post-operatively achieved mLDFA compared to intended mLDFA-goal showed a mean of 0.14° ± 0.56° SD (95% CI: -0.26° to 0.54°). When both groups were compared based on their post-operative achieved mLDFA (87-goal-group versus 90-goal-group), two sample Wilcoxon (Mann-Whitney) test showed a significant difference (*p* = 0.008). Detailed information is given in Table 1.

**Table 1:** Results of measurements, mean and SD, values in degrees

		Preoperative			Calculations			Postoperative	
		Torsion	mLDFA	AMA at cut	Corrective angle	Resulting corrective angle	Oblique cutting angle	Torsion	mLDFA
87-goal-group	mean	41.24	82.92	5.396	4.68	3.44	10.14	21.52	86.86
	SD	4.75	1.88	1.15	2.08	2.04	6.10	5.22	0.66
90-goal-group	mean	32.88	85.98	6.634	4.6	3.54	10.44	13.22	90.42
	SD	6.08	1.27	0.89	1.37	1.57	4.66	6.63	0.25
All specimens combined	mean	37.06	84.45	6.015	4.64	3.49	10.29	17.37	
	SD	6.77	2.21	1.17	1.66	1.72	5.12	7.12	

The mean sagittal inclination of the cutting plane was 10.3° (range 3.5° – 19.3°). Ten cutting guides were 3D-printed, giving an individual oblique cutting plane from antero-proximal to postero-distal from a sagittal point of view. Duration of 3D-printing took approximately 60 min and had a filament cost of about $0.50 per guide. The doubled postoperative measurements showed an averaged standard deviation of ±0.25°.

### Mathematical model

**Fig. 5:**
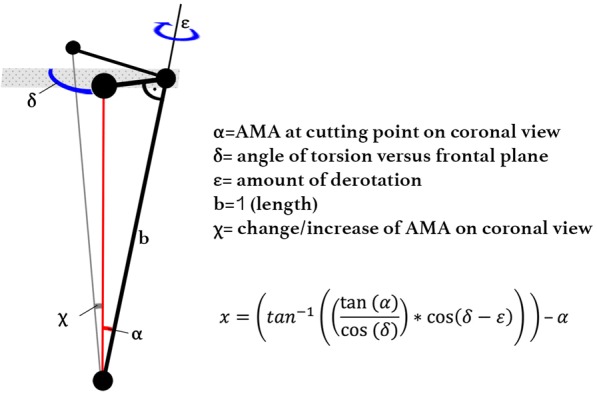
Illustration and Equation for change of AMA at cutting point

Trigonometric calculations showed varus producing effects after a simple internal rotation of the proximal limb when the cutting plane was 90 degrees to the proximal virtual anatomical axis (regarding the greater trochanter) [22]. Table 2 shows the change of AMA at a defined torsion angle, which is independent from limb length, (formula is shown in Fig. 5 in Appendix).

**Table 2:** The AMA table; a certain antetorsion and an intended derotation will give a specific additional change on AMA at a specific measured AMA, reprinted from Imhoff et al. [22]

Antetorsion (°)	25	30	30	35	35	40	40	40	45	45	45
Derotation (°)	10	10	15	15	20	20	25	30	20	25	30
AMA (°)											
3	0.2	0.3	0.3	0.4	0.5	0.7	0.8	0.9	0.8	1.0	1.1
3.5			0.4	0.5	0.6	0.8	0.9	1.0	1.0	1.1	1.3
4	0.3	0.3	0.5	0.6	0.7	0.9	1.0	1.1	1.1	1.3	1.5
4.5			0.5	0.7	0.8	1.0	1.2	1.3	1.3	1.5	1.6
5	0.3	0.4	0.6	0.7	0.9	1.1	1.3	1.4	1.4	1.6	1.8
5.5			0.6	0.8	1.0	1.2	1.4	1.6	1.5	1.8	2.0
6	0.4	0.5	0.7	0.9	1.1	1.3	1.5	1.7	1.7	2.0	2.2
6.5			0.7	0.9	1.2	1.5	1.7	1.8	1.8	2.1	2.3
7	0.5	0.6	0.8	1.0	1.2	1.6	1.8	2.0	1.9	2.3	2.5
7.5			0.9	1.1	1.3	1.7	1.9	2.1	2.1	2.4	2.7
The AMA table				Change of AMA (°) = varus increase							

**Fig. 6:**
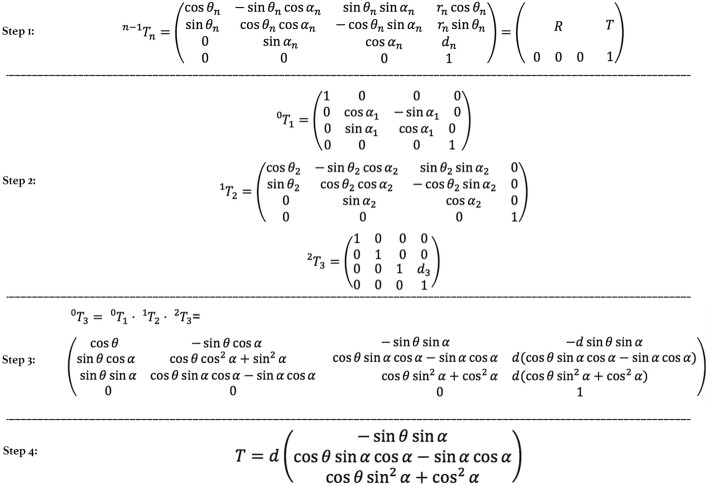
Mathematical equation, Denavit-Hartenberg Transformation: Development of equation for change of axis due to rotation and an oblique cutting angle

**Fig. 7:**
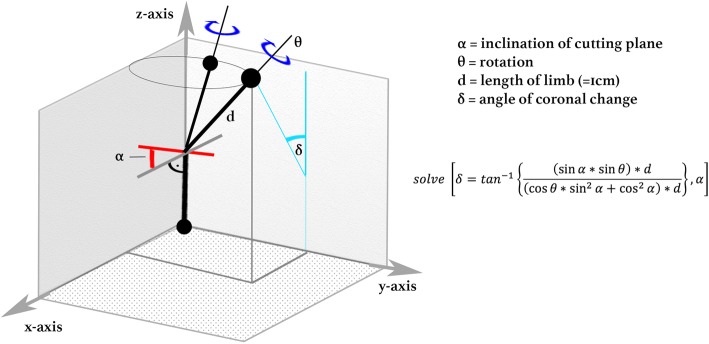
Illustration of schematic XYZ-coordinate for calculations of three dimensional effects; reverse equation for angle of coronal change (XZ-plane)

The inclination of the cutting plane was calculated with the use of the Denavit-Hartenberg notation (equations are shown in Figs. 6 and 7 in Appendix). In order to receive a significant (*p* = 0.0001) change of axis on the coronal view, inclination of the cutting plane had to be angled from a sagittal point of view (varus producing: anterior-proximal to posterior-distal, valgus producing: anterior-distal to posterior-proximal), if perpendicular from a coronal view, when internal rotation of the proximal limb (external rotation of the distal limb) is performed (Table 3). In addition to the change on frontal axis, the simultaneous change of the axis from a sagittal view (extension/flexion of the femur) is given in parentheses and shows only marginal change on axis. An example of calculations with the specimen’s mean values is given in the Appendix. According to the mathematical model, length of the limb changed with a shortening by only 0.2% when derotation of 20° is performed at an inclined cutting angle of a mean of 10.3°.

**Table 3:** The robotic table: An intended derotation angle and an intended change on the coronal axis, equals a specific inclined cutting angle from the sagittal view

Change on coronal axis	Derotation = external rotation of the distal limb / internal rotation of proximal limb							
	10°	15°	20°	25°	30°	35°	40°	
1	5.8	3.9	2.9	2.4	2.0	1.7	1.6	*Additional change on sagittal axis in []*
1.5	8.7	5.8	4.4	3.6	3.0	2.6	2.3	
2	11.6 [0.2]	7.8	5.9	4.7 [0.4]	4.0	3.5	3.1 [0.7]	
2.5	14.5	9.7	7.3	6.0	5.0	4.4	3.9	
3	17.5	11.7	8.8	7.1	6.0	5.2	4.7	
3.5	20.6	13.6	10.3	8.3	7.0	6.1	5.4	
4	23.7 [0.3]	15.6	11.8	9.5 [0.9]	8.0	7.0	6.2 [1.4]	
4.5	26.9	17.6	13.3	10.7	9.0	7.9	7.0	
5	30.1	19.7	14.8	11.9	10.0	8.7	7.8	
5.5	33.5	21.7	16.3	13.1	11.0	9.6	8.6	
6	37.0 [0.4]	23.8	17.8	14.3 [1.3]	12.1	10.5	9.4 [2.2]	
6.5	40.7	26.0	19.3	15.5	13.1	11.4	10.1	
7	44.6	28.1	20.9	16.8	14.1	12.3	10.9	
7.5	48.7	30.3	22.4	18.0	15.1	13.1	11.7	
8	53.3 [0.4]	32.5	24.0	19.2 [1.7]	16.2	14.0	12.5 [2.7]	
8.5	58.4	34.8	25.6	20.5	17.2	14.9	13.3	
9	64.3	37.2	27.2	21.7	18.2	15.8	14.1	
9.5	71.9	39.6	28.8	23.0	19.3	16.7	14.9	
10	89.0 [.01]	42.1	30.5	24.3 [2.0]	20.3	17.6	15.6 [3.5]	
The Robotic Table	Inclination of cutting plane from sagittal view							All values in degrees (°)
	Ant.-prox. to post.-dist. = varus producing							
	Ant.-dist. to post.-prox. = valgus producing							

## Discussion

The most important findings of the study were that when a pre-calculated inclined osteotomy is performed, which is referred to the virtual anatomical axis by use of a simple 3D printed cutting guide, a precise postoperative result on frontal and torsional angle can be achieved in distal femur rotational osteotomy. The objective of this study was to develop simplified charts for angle calculations based on precise mathematical models and provide a proof of concept in a cadaveric model to demonstrate the predictability of 3D correction in the clinical setting with the use of simple individual cutting guides.

Derotational osteotomy is the gold standard for the treatment of torsional deformities in the lower extremity, as Dickschas et al. postulate by regards of their clinical cases [4]. Little is known about correlation of valgus malalignment and patellofemoral instability. According to the literature, combining a distal femoral lateral open-wedge or medial closing-wedge osteotomy to a derotational osteotomy is rarely done because of its complexity [7]. But, several studies suggest that increased valgus deformity is considered to be an additional risk factor in such cases [4, 7, 24]. Furthermore, different studies have shown the correlation of valgus alignment and increase of patellofemoral arthritis [25–28]. Yet it is unknown if correction of valgus malalignment may necessarily correct tracking abnormalities at the patellofemoral joint [28]. The proposed Q-angle by Brattström in 1964 and his annotations grounded the idea of alignment tracking [29]. He suggested that muscular vectors might be improved due to derotation, although the Q-angle may not be reliable and very accurate in clinics [30]. We believe that a combined correction of alignment, which is a derotation of an increased antetorsion and correction of valgus malalignment, improves patellar stability and patellar tracking. Therefore, our single cut approach in distal femoral derotational osteotomy can avoid aggravating frontal malalignment and can even be used for an intended change on axes.

Single-cut correction for torsional and angular deformity has been described many years ago by the French D’Aubigne in 1952 [31]. There is even a European patent from 1996 held by a French inventor Du Toit for surgical instruments to “guide a saw while it makes an oblique cut in a long bone” (EP 0570187). Subsequently the planning of the osteotomy angle with mathematical models and tables was improved in the 80’s for example by Sangeorzan et al. [32] Despite the mathematical calculations to achieve the correct osteotomy plane, Gürke and Strecker et al. showed a graphical approach to define the plane of the single-cut osteotomy in 1999 [33]. However, our approach does not address the center of deformity and we aimed to achieve a reproducible technique for cases of patellofemoral instability and indicated derotational osteotomy. Therefore, only one plane is inclined, given by an easy-to-read table, from the lateral view versus the shaft, as this reflects the standard surgical approach.

It is known that any rotational osteotomy will have changes on frontal and sagittal axes as described by Paley et al. in principles of deformity corrections, and as shown by Kim et al. and Lee et al. in their computed mathematical articles [15, 16, 34]. In order to receive reproducible results in a clinical cadaver model, a new mathematical approach for calculation of the cutting plane was chosen. Our findings show that a correct reference of the cutting plane versus the anatomical shaft is elementary. Using a cutting plane perpendicular to the virtual anatomic axis will lead to a slight increase of AMA and will not aggravate a valgus malalignment. Trigonometrical calculations from the robotics show that inclination of the cutting plane from the sagittal view will have the most change on axis on the coronal view, and only slight change of axis on the sagittal view. Based on the presented table (Table 3), change on varus (cutting plane: antero-proximal to postero-distal) or valgus (cutting plane: antero-distal to postero-proximal) alignment can be performed.

This presented “robotic formula” and the provided table (Table 3) with common values for clinical use show that an inclination of the cutting plane between 8.8° and 11.8° (3° difference) will have a change in the coronal change of axis from 3.5° to 4° (0.5° difference), when rotation by 20° is performed. In terms of clinical accuracy this may be acceptable; however, this can be improved by more exact surgical guides as it is shown in this study and has been described for distal femur open wedge osteotomy by Victor et al. in 2013 [35].

The current study may help to explain why reference of the osteotomy plane to the shaft is one of the most important step in such procedures in order to avoid unintended changes on axes. Bowing of the femur can lead to increased or decreased mLDFA, dependent whether osteotomy is proximal or distal as Nelitz et al. showed in a computed model [1]. In that study, the cutting plane was always set to be perpendicular to the actual anatomic shaft, which differs distally versus proximally. We believe that our approach is able to simplify derotational osteotomy and to avoid postoperative malalignment. Angulation of the cutting plane to the virtual anatomical axis is key to an exact reproducible result in our study.

It is likely that other factors lead to a certain margin of error when measuring angles. To improve accuracy in calculations, an exact frontal plane radiograph of the knee joint is important. As coronal CT reconstructions may help to improve accuracy regarding correct plane of views, we normally perform MRI images for torsion measurement to reduce radiation to young patients. Methods for torsion measurement are well investigated and show reliable results in terms of intraobserver and interobserver agreement as Kaiser et al. describe [36]. But different threshold values depending on the measurement technique should be considered in clinical use. We used the method from Waidelich et al. for exact pre- and postoperative measurements because of the iatrogenic prepared proximal femur bones [19]. These had passed through different femoral neck removals due to the THA workshop, but fully preserved femoral heads and trochanters. Therefore, this method was suitable for our model, and with regards to the virtual anatomical axis, this method supports our model even more.

The performed derotation of 20°, which is visually controlled by two k-wires, shows acceptable results (mean 19.69°). Regarding the mathematic formula, if cutting plane is 10° oblique, derotation between 19° and 21° results in a difference of change of coronal axis by only 0.4°. In order to gain perfection for rotational control, an electromagnetic tracking device processing in real-time as Geisbuesch et al. describe, could be added [37]. The height of the osteotomy (7 cm from the distal joint line) which was chosen due to plate design should not affect the overall outcome, if planning of the osteotomy with its corrective angle is done at this same height. If the osteotomy is supposed to be more proximal, the corrective angle will increase and the inclination of the cut will have to be increased, as well. The mathematical model does not have an angular limitation. However, as shown in Table 3 (the robotic table), a derotation of 20° and an accompanied frontal alignment correction of 10° equals an oblique cutting angle of 30°, which has to be considered in practice.

The principal limitations of this work lie in the perfect anatomical overview from the frontal and lateral view in order to navigate the perfect osteotomy cut. The removed soft tissue helped to perfectly rotate and handle the osteotomy. In vivo, this can be very challenging regarding medial-lateral translation or missed hinge of the osteotomy. The biomechanical nature of the study contains iatrogenic prepared specimen, which showed common pathologic angle values (increased torsion and slight decreased mLDFA). We produced antetorsion artificially in the femoral neck after THA by k-wire fixation. However, the problem of antetorsion of the femur is not only located at the femoral neck. It can occur at the proximal, diaphyseal or distal side of the femur, which was shown by Seitlinger et al. [18] Moreover, this simplified model does not involve any form of dysplasia of the condyles, trochlea, and shaft, which may be observed in patellofemoral instability cases. Another limitation is that surgery was performed by only one surgeon, in order to receive consistent data and proof the concept. But when the calculations and surgical approach are taken into clinics and performed by several surgeons, accuracy may be decreased. 3D-printed cutting guides are used in several publications, and even in osteotomy surgery [35, 38, 39]. But, its practicality and understanding are not widespread. Therefore, stainless steel cutting guide assemblies with intraoperative adjustments of the inclination may help surgeons as well. For a consecutive study, we suggest to perform the analysis and surgery on total leg cadavers with soft tissue for proof of its feasibility in a clinical setup.

## Conclusion

A preoperative calculated angle for single cut derotational osteotomy at the distal femur led to a clinically precise post-operative result on torsion and frontal alignment when using this approach. 3D-changes were mathematically predicted and provided by the use of an individualized cutting guide, based on angle measurements from standard two-dimensional radiograph and axial CT slicing.

## Appendix

### Example calculation of cutting angle (mean values of specimens)

Each specimen underwent specific radiographic analysis, giving an intended mean corrective angle of 4.64° observed on frontal plane x-ray, and a mean change of AMA at cutting point of 1.15° when derotation of 20° was intended. This results in a mean remaining corrective angle of 3.49°. Converting the remaining corrective angle resulted in a mean sagittal inclination of the cutting plane of 10.06°. Aside from intended change on the coronal view (increased varus), additional calculations showed a mean simultaneous change on the sagittal view (increased extension) of only 0.59° (*p* = .0001).

### Change of AMA at cutting

Trigonometrical calculations were done to forecast the angular changes on “AMA at cutting point” when internal rotation of the proximal limb is performed at a cutting plane perpendicular to the virtual anatomical shaft from a coronal and a sagittal view. No actual lengths are needed when calculations are done with at least two known angles in a perpendicular triangle (Fig. 5 in Appendix) [22].

### Denavit-Hartenberg Transformation

In order to calculate the position of the rotated end point after definition of a cutting plane and a rotation angle, an approach commonly used in from robotics was applied [17]. Hereby, a combination of translational and rotational matrices was used to calculate the position of a robot joint. The coordinates *x*_*n*_, *y*_*n*_, *z*_*n*_ of a joint number *n* can be calculated using a standardized transformation of the coordinate system of the joint number *n* − 1 with the axes *x*_*n* − 1_, *y*_*n* − 1_, *z*_*n* − 1_. The Denavit-Hartenberg transformation matrix for the transition from position *n* − 1 to position *n* can be described (Fig. 6 in Appendix, Step 1):

Here, *d*_*n*_ describes a translation along the original *z*_*n* − 1_ axis. The angle *θ*_*n*_ defines a rotation around the original *z*_*n* − 1_ axis, from original *x*_*n* − 1_ to new *x*_*n*_. The distance *r*_*n*_ denotes an offset perpendicular to the original *z*_*n* − 1_ axis, which can be set to zero in this case. Finally, the angle *α*_*n*_ describes a rotation around the new *x*_*n*_ axis.

In the particular case presented in this paper, the following three transformations are required to describe the position of the rotated end point relative to the center of the rotational plane: At first, the angle *α*_1_ ≡ *α* is used to define the cutting plane. Subsequently, the actual rotation around the cutting plane is described by *θ*_2_ ≡ *θ*, followed by a reverse rotation around *α*_2_ =  − *α*_1_ =  − *α* to account for proper positioning of the end point with respect to the cutting plane. Finally, the translation *d*_3_ ≡ *d* denotes the length of the piece to be rotated, i.e. the distance of the rotated end point from the rotational plane.

Hence, the three respective transformation matrices can be constructed (Fig. 6 in Appendix, Step 2). The final transformation matrix is the result of the three matrices multiplications (Fig. 6 in Appendix, Step 3). The cartesian position of the rotated end point is given by the first three row elements of the fourth column (Fig. 6 in Appendix Step 4). When given a defined inclination angle and a defined rotation angle, resulting changes on XYZ-axis can be calculated in distance (Fig. 7 in Appendix). Adding trigonometric equations will also help calculate angles for coronal (XZ-plane) and sagittal (YZ-plane) changes of axes.

## Abbreviations

3D: Three-dimensional
aLDFA: Anatomical lateral distal femur angle
AMA: Anatomical mechanical axis
mLDFA: Mechanical lateral distal femur angle
